# Algorithm for Automatic Rod Feeding and Positioning Error Compensation for Underground Drilling Robots in Coal Mines

**DOI:** 10.3390/s23177530

**Published:** 2023-08-30

**Authors:** Qianhai Lu, Lingfei Kong, Guangyu Peng, Wang Jia, Sun Jin, Chenyu Dai, Qianxiang Zhu

**Affiliations:** 1 School of Mechanical and Precision Instrument Engineering, Xi’an University of Technology, Xi’an 710048, China; 2 CCTEG Xi’an Research Institute (Group) Co., Ltd., Xi’an 710077, China

**Keywords:** drilling robot, compensation algorithm, calibration, positioning error, automation

## Abstract

In the pursuit of automating the entire underground drilling process in coal mines, the automatic rod feeding technology of drilling robots plays a crucial role. However, the current lack of positional accuracy in automatic rod feeding leads to frequent accidents. To address this issue, this paper presents an algorithm for compensating positioning errors in automatic rod feeding. The algorithm is based on a theoretical mathematical model and manual teaching methods. To enhance the positioning accuracy, we first calibrate the pull rope sensor to correct its measurement precision. Subsequently, we establish a theoretical mathematical model for rod feeding positions by employing spatial coordinate system transformations. We determine the target rod feeding position using a manual teaching-based approach. Furthermore, we analyze the relationship between the theoretical rod delivery position and the target rod delivery position and propose an anisotropic spatial difference compensation technique that considers both distance and direction. Finally, we validate the feasibility of our proposed algorithm through automatic rod feeding tests conducted on a coal mine underground drilling robot. The results demonstrate that our algorithm significantly improves the accuracy of rod feeding positions for coal mine underground drilling robots.

## 1. Introduction

The coal mining industry is crucial for the national economy [1] but faces significant challenges [2], including water damage control [3]. Conventional underground drilling operations in coal mines heavily rely on manual labor, leading to high safety risks and low productivity [4]. To overcome these limitations, researchers have focused on developing coal mine underground drilling robots [5,6,7]. These intelligent robotic systems can autonomously perform drilling tasks [8], utilizing advancements such as automatic attitude adjustment, autonomous navigation, sensing, and control [9,10,11,12,13,14]. Among these advancements, automatic rod feeding is a critical technology for coal mine underground drilling robots. It allows the robots to deliver drill rods to designated positions without manual intervention [15]. However, practical rod feeding operations often encounter significant errors, leading to unsuccessful rod feeding operations. Therefore, it is essential to enhance the precision of the rod feeding position to reduce the failure rate of automatic rod feeding operations.

In order to enhance the positional accuracy of robot automatic feeding, numerous scholars [16,17,18,19] have carried out relevant technical research. Yuanfan Zeng [20] proposed a method to improve the positioning accuracy of the drilling and riveting system by utilizing error similarity. This approach establishes an error model based on the spatial similarity of robot joints and estimates the positional error of the target. The method effectively enhances the absolute positioning accuracy of industrial robots and meets the requirements of positioning accuracy for robot drilling and riveting systems. Nguyen Van Toan [21] presented a kinematic calibration method based on the singular value decomposition least squares algorithm (SVD-POE-Least Squares algorithm) of the exponential product formula. This method addresses the problem of calibration algorithms failing to converge and exhibiting low accuracy in the presence of environmental noise. By employing this approach, the accuracy of the robot’s kinematic calibration is significantly improved. Dongdong Chen [22] proposed a cooperative kriging-based error compensation method, which primarily relies on measurement data to fit the position error cross-variance function. This method utilizes the error similarity of robot kinematics, estimates the predicted position error using cooperative kriging, and, ultimately, compensates for positioning errors. This technique effectively enhances the positioning accuracy of aerial robots. Although these approaches focus on error compensation from the perspective of the robot’s kinematic accuracy, several other scholars [23,24] have conducted extensive research in this area. While these algorithms have contributed to improving the positioning accuracy of robots to some extent, their efficacy is limited and requires extensive experimental validation. In order to enhance positional accuracy, researchers have explored the usage of advanced vision systems to compensate for the robot’s positioning error [25,26]. Some notable approaches in this regard include the following: Biao Mei [27] proposed a two-position vision system that measures the R-S coordination error margin and develops a corresponding control system. This system includes in-process points, coordination error measurement, and drilling position correction, effectively improving the robot’s positioning accuracy. However, it should be noted that this system is more expensive and requires a more advanced machine vision system. Yufei Li [28] introduced an area compensation method specifically designed for industrial robots with TCP calibration uncertainty. This method addresses localization accuracy in scenarios where calibration uncertainty and significant changes in the TCP direction are present. However, the applicability of this method is limited and primarily suitable for machining fields. Qiang Zhan [29] presented a robot drilling system based on hand–eye calibration and localization. This approach describes the position of the target point in the robot coordinate system through the hand–eye relationship between the robot coordinate system and the vision coordinate system, utilizing calibrated hand–eye relationships. This resolves inconsistencies between the mathematical model and the localization position, resulting in enhanced robot localization accuracy. While all of these methods employ machine vision technology to further enhance positioning accuracy, it is important to note that the application of vision systems in coal mine underground tunnels faces challenges. These spaces are characterized by narrow dimensions and insufficient lighting, making the deployment of vision systems difficult. In the field of drilling robots, error compensation models are commonly employed to enhance localization accuracy. Xuanqi Zhou [30] has proposed a novel approach by combining both geometric and non-geometric error compensation methods. They designed a parallel differential evolutionary algorithm based on the kinematic model and introduced a hybrid positional error compensation method that utilizes the Radial Basis Function Network (RBFN) and the Light Gradient Advancement Decision Tree (LightGBM). This method proves effective in improving the control accuracy of rock drilling robots, significantly reducing labor intensity, and enhancing the efficiency and quality of tunneling operations. In a similar context, Y Xia [31] has developed an error compensation kinematic model for the drill rod, which is based on the DH kinematic model and encompasses the dynamic and static errors of the drill arm. The approach involves the utilization of migration operators, artificial selection operators, and multiple swarm genetic algorithms to establish a positioning algorithm for the drill pipe, leveraging the kinematic error compensation model. The aforementioned algorithms successfully achieve the desired positioning functionality. However, it is important to note that the algorithm complexity, as well as various influencing factors in engineering applications, result in a less satisfactory real-world performance. In comparison to other drilling robots, coal mines present additional challenges due to the presence of flammable gases. Therefore, all equipment used in coal mines must possess intrinsic safety and explosion-proof capabilities [32,33]. This requirement limits the usage of common advanced equipment and technologies that lack the necessary explosion-proof function. Consequently, the automatic rod feeding technology becomes a technical bottleneck in the intelligentization of drilling robots within coal mines. Furthermore, the working range of coal mine underground drilling robots encompasses a substantial space. Ensuring the positional accuracy of the automatic feed rod for all points within this extensive workspace to align with operational requirements presents yet another technical challenge. Hence, there is an urgent need to design a simple and feasible error compensation method that can enhance the positioning accuracy of drilling robots operating under these specific conditions.

This paper presents a novel automatic rod feeding positioning error compensation algorithm based on manual teaching and mathematical modeling. The algorithm aims to address the problem of significant deviations between the theoretical and target rod feeding positions, which often result in the failure of rod feeding operations in coal mine underground drilling robots. By implementing this algorithm, the positioning accuracy of coal mine underground drilling robots can be improved.

The organization of the paper’s chapters is as follows:

Section 2 introduces the composition of the downhole drilling robot used in coal mines and presents a theoretical mathematical model for automatically determining the rod feeding position.

Section 3 examines the potential errors associated with the automatic rod feeding position and proposes compensation methods, along with an error compensation model.

Section 4 focuses on the construction of an experimental platform designed for sensor calibration and correction. This platform facilitates the verification of the spatial difference error compensation algorithm.

In Section 5, the algorithm undergoes rigorous validation through field drilling tests, successfully meeting the requirements of the drilling process.

Finally, Section 6 concludes the paper by summarizing the key findings and research contributions.

## 2. Methods

### 2.1. Introduction to Underground Drilling Robots in Coal Mines

**Figure 1:**
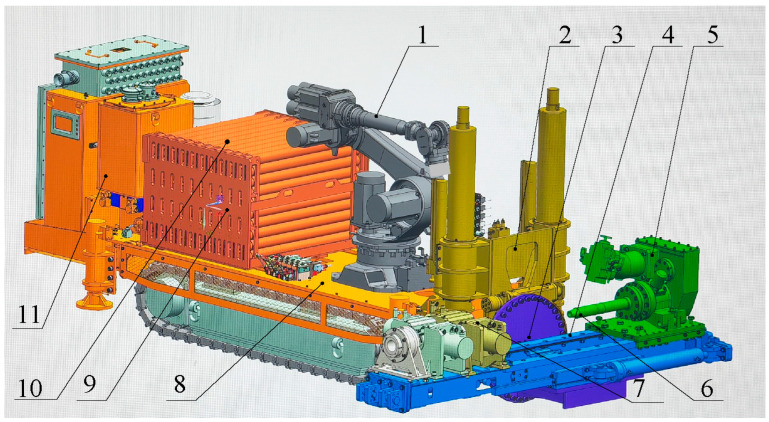
Three-dimensional diagram of coal mine underground drilling robot. 1. Industrial robot; 2. column lifting device; 3. rotating platform; 4. translating feeding device; 5. power head; 6. active drilling rod; 7. buckle unloader; 8. crawler body platform; 9. drilling rod box; 10. drilling rod; 11. control center.

The coal mine underground drilling robot is equipped with several essential functionalities including remote-controlled movement, precise positioning, and automatic loading and unloading of drill pipes. Figure 1 illustrates a three-dimensional structure diagram of the coal mine underground drilling robot. The crawler body platform supports the column lifting device, which facilitates the vertical movement of the rotating platform. The rotating platform has the capability to rotate around its center of rotation. Furthermore, a translation feeding device is installed on the rotary platform, enabling the power head, detent, and other mounted components to undergo translational motion along the mounting surface of the rotating platform. The power head can generate relative translational movement on the translation feeding device. Meanwhile, the detent is permanently fixed onto the translation feeding device.

During the operation of the underground coal mine drilling robot, the control center oversees the movement of the lifting device, rotating platform, and translating feeding device to position them appropriately. This positioning allows for the execution of opening operations at varying heights and inclinations. Following this, the drilling construction operation takes place. Before each drill pipe is automatically fed, the height lifting sensor, translation feeding sensor, and angle sensor collect the opening position data (L, H, θ). Subsequently, the automatic feeding position algorithm in the industrial control computer calculates the theoretical feeding position. The industrial robot receives this information and retrieves the drill pipe from the drill pipe box. It then places the drill pipe into the theoretical feeding position between the unloader and the active drill pipe. This process enables the automatic loading of the drill pipe. In contrast, the unloading operation is performed similarly but in reverse. Before each drill pipe is removed, the Industrial Control Module (ICM) calculates the theoretical unloading position based on the sensor measurement data. The industrial robot moves to the designated unloading position, extracts the drill pipe, places it back into the drill pipe box, and completes the unloading operation.

### 2.2. Mathematical Model of Underground Drilling Robot in a Coal Mine

**Figure 2:**
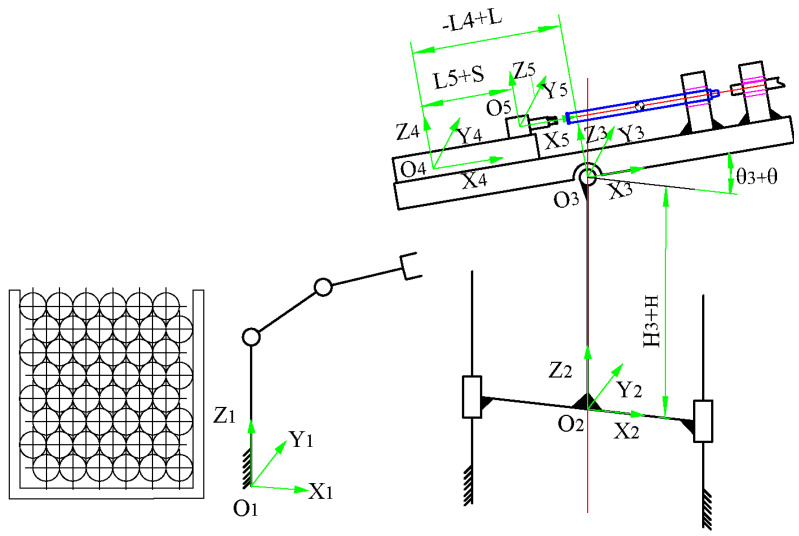
Schematic representation of the main structure motion of an underground coal mine drilling robot.

Figure 2 represents a sketch of the motion structure of the coal mine underground drilling robot, which is mathematically modeled according to the relationship between coordinate systems, where *O_w_-X_w_Y_w_Z_w_* is the world coordinate system, *O*_1_*-X*_1_*Y*_1_*Z*_1_ is the industrial robot coordinate system centered on the bottom of the industrial robot base, which overlaps with the world coordinate system, *O*_2_*-X*_2_*Y*_2_*Z*_2_ is the column lifting device, *O*_3_*-X*_3_*Y*_3_*Z*_3_ is the rotating platform coordinate system, *O*_4_*-X*_4_*Y*_4_*Z*_4_ is the translating feed device coordinate system, *O*_5_*-X*_5_*Y*_5_*Z*_5_ is the power head coordinate system, and *O*_5_*-X*_5_*Y*_5_*Z*_5_ is the rotating platform coordinate system. *O*_3_*-X*_3_*Y*_3_*Z*_3_ is the rotating platform coordinate system, *O*_4_*-X*_4_*Y*_4_*Z*_4_ is the translation feed device coordinate system, and *O*_5_*-X*_5_*Y*_5_*Z*_5_ is the power head coordinate system. The transformation matrix from the world coordinate system to the power head coordinate system can be expressed as follows:

(1) T5C=T51=T21×T32×T43×T54

where each transformation matrix can be expressed as follows:

(2) Tii−1=Trans(ai,0,0)×Rot(αi,0,0)×Trans(0,bi,0)×Rot(0,βi,0)×Trans(0,0,ci)×Rot(0,0,γi)

In the given context, let *a_i_*, *b_i_*, and *c_i_* represent the distances between the ith coordinate system and the (i-1)st coordinate system in the *x*, *y*, and *z* directions, respectively. Additionally, *α*, *β*, and *γ* represent the rotation angles around the *z*, *y*, and *z* axes, respectively. The transformations involved in this scenario include Trans(-) and Rot(-), which denote the translation and rotation matrices, respectively.

(3) $$Trans(x,y,z)=\left[\begin{array}{cccc} 1 & 0 & 0 & a_{i}\\ 0 & 1 & 0 & b_{i}\\ 0 & 0 & 1 & c_{i}\\ 0 & 0 & 0 & 1 \end{array}\right]$$

(4) $$Rot(\alpha ,\beta ,\gamma )=\left[\begin{array}{cccc} \cos(\alpha )\cos(\beta )\cos(\gamma )-\sin(\alpha )\sin(\gamma ) & -\cos(\alpha )\cos(\beta )\sin(\gamma )-\sin(\alpha )\cos(\gamma ) & \cos(\alpha )\sin(\beta ) & 0\\ \sin(\alpha )\cos(\beta )\cos(\gamma )+\cos(\alpha )\sin(\gamma ) & -\sin(\alpha )\cos(\beta )\sin(\gamma )+\cos(\alpha )\cos(\gamma ) & \sin(\alpha )\sin(\beta ) & 0\\ -\sin(\beta )\cos(\gamma ) & \sin(\beta )\sin(\gamma ) & \cos(\beta ) & 0\\ 0 & 0 & 0 & 1 \end{array}\right]$$

Table 1 presents the relationship between the size and angle parameters of the components comprising the ZDY4500LK underground drilling robot. The parameters include the following:

1. *H*: Displacement of the column lifting device, ranging from 0 to 400 mm.
2. *L*: Displacement of the translation feeding device, ranging from −440 mm to 440 mm.
3. *S*: Displacement of the power head, which needs to provide sufficient space for the drill pipe. In order to achieve this, the power head must be moved to the furthest end face. Therefore, S is set to 0.
4. *θ*: Angle value of the rotation of the rotating platform, ranging from −90° to 90°.

In the coordinate system of the power head, the center point coordinate of the target feeding position is denoted as *p* (*m*, 0, *n*). Here, *m* corresponds to 407 mm, and *n* corresponds to 260 mm. The expression used for theoretically calculating the center point coordinate of the target feeding position in the world coordinate system is as follows:

(5) pw=T5w×p5==cos(j)0sin(j)(a4+a5)×cos(j)+(c4+c5)×sin(j)010b2−sin(j)0cos(j)c2+c3−(a4+a5)×sin(j)+(c4+c5)×cos(j)0001×m0n1

The parameters can be obtained by bringing them into Equation (5):

(6) $$p=\left[\begin{array}{c} x\\ y\\ z \end{array}\right]=\left[\begin{array}{c} 260\times \sin(j)+(201+L)\times \cos(j)\\ 1345\\ 260*\cos(j)+540+H-(201+L)\times \sin(j) \end{array}\right]$$

The industrial robot employs the *z-y-z* transformation in conjunction with the manipulator’s installation to calculate the Euler angles using the *z-y-z* sequence. Specifically, when the rotating platform rotates by an angle of *θ*, denoting *j* = *−θ*, the Euler angle of the corresponding industrial robot can be obtained.

In cases where *θ* is greater than 0,

(7) $$\left\{\begin{array}{c} A=0\\ B=180-\left|\theta \right|\\ C=90 \end{array}\right.$$

When *θ* < 0,

(8) $$\left\{\begin{array}{c} A=-180\\ B=180-\left|\theta \right|\\ C=-90 \end{array}\right.$$

When *θ* = 0,

(9) $$\left\{\begin{array}{c} A=-90\\ B=180\\ C=0 \end{array}\right.$$

## 3. Error Compensation for Underground Drilling Robots in Coal Mines

During the delivery of rods by the underground drilling robot in coal mines, a theoretical model of the robot is typically used to calculate the theoretical delivery position parameters. Subsequently, an industrial robot is employed to grasp the rods and transport them to the intended delivery position. However, practical implementation reveals that the underground drilling robot frequently encounters difficulties in delivering the drill pipe successfully. It has been observed that the actual position consistently deviates from the desired axis alignment with the center axis of the power head, active drill pipe, and unbuckler. Consequently, a significant deviation exists between the actual feed position and the target feed position, leading to unsuccessful feed operations. In certain instances, this misalignment may result in collisions between the drill pipe and either the buckler or the active drill pipe, potentially causing damage to the underground drilling robot within the coal mine.

### 3.1. Error Analysis

Based on the actual working conditions and environment of the drilling robot operating underground in coal mines, a theoretical analysis was conducted to identify the primary factors contributing to the deviation observed between the actual and target rod delivery positions. The following are the main reasons determined:

(1) Sensor accuracy issue: The sensor utilized by the drilling robot lacks the necessary precision to provide accurate position information, resulting in deviations in the actual rod feeding position of the underground coal mine drilling robot.
(2) Mechanical processing and assembly inaccuracies: During the manufacturing process, various degrees of errors occur in the fabrication of parts and components for the underground coal mine drilling robot. Additionally, assembly errors arise during the construction of the robot, particularly with larger parts. While it may not be possible to completely eliminate these errors, efforts are made to minimize their impact.
(3) Positioning and placement errors of the flexible hand claw: The flexible hand claw incorporates a certain level of flexibility, which reduces the strictness of rod positioning requirements for the underground coal mine drilling robot. However, this flexibility also contributes to decreased accuracy in rod positioning.

The sensor accuracy error can be rectified through calibration post-installation, thus enhancing the sensor’s detection accuracy. Once the assembly of the coal mine underground drilling robot is finalized, the machining and assembly errors, along with the flexible gripper positioning and placement errors, are essentially established as fixed values. These errors are categorized as internal errors inherent to the coal mine underground drilling robot. However, they can be compensated for by employing pertinent algorithms to enhance the accuracy of the feeder position.

### 3.2. Sensor Error Compensation

After the installation of sensors, it is crucial for the coal mine underground drilling robot to verify and correct any sensor errors to ensure accurate measurements. The height elevation sensor, translation feed sensor, and angle sensor used in this study are all pull rope sensors. When subjected to different lengths or angles of deformation, the rope sensor utilizes a metal elastomer to convert this deformation into an electrical signal output. By reading the output signal and applying Hook’s law, the signal is further converted into a measured value, determining the length or angle.

When using sensors, it is common to observe a discrepancy between the theoretical measurement value and the actual value, which has a negative impact on measurement accuracy. Therefore, it is necessary to perform corrections. In the case of underground drilling robots, it is crucial to adjust the length coefficients of the height elevation sensor, the translation feed sensor, and the angle coefficients of the angle sensor. These coefficients can be determined by comparing the differences between the actual measured length or angle values and the corresponding differences in the number of sensor rotations. This correction process enables the refinement of sensor coefficients and the verification of measurement accuracy. The specific steps involved in the correction process are outlined below.

The formula for measuring the length of a pull cord sensor Is as follows:

(10) $$L=\Delta L+L_{0}$$

where *L* is the measured length, Δ*L* is the deformation of the pull rope, and *L*_0_ is the zero length.

The formula for Δ*L* is as follows:

(11) $$\Delta L=k\times (n-n_{0})$$

where *k* is the length coefficient, *n* represents the number of pull rope turns recorded by the pull rope sensor during measurement, and *n*_0_ represents the number of pull rope turns when the pull rope sensor is at zero length.

The primary objective of validating the pull rope sensor is to adjust the length coefficient, *k*, to ensure that the measurement value of the sensor aligns with the actual value. Assuming that the actual lengths are *L*_1_ and *L*_2_, and their corresponding number of pull rope sensor turns are *n*_1_ and *n*_2_, respectively, the corrected length coefficient, *k*_l_’, can be calculated as follows:

(12) $$k_{l}\prime =\frac{L_{2}-L_{1}}{n_{2}-n_{1}}$$

The principle for measuring length and angle using the pull rope sensor is similar, and, therefore, the same rationale applies. Following the adjustment of the pull rope sensor, the angle coefficient, *k_θ_*’, can be determined using the following formula:

(13) $$k_{\theta }^{\prime }=\frac{\theta_{2}-\theta_{1}}{n_{2}-n_{1}}$$

where *θ*_1_ and *θ*_2_ represent the actual angle values, while *n*_1_ and *n*_2_ correspond to the respective number of turns recorded by the pull cord sensor.

### 3.3. Feeder Position Error Compensation

#### 3.3.1. Operational Range of the Underground Drilling Robot in Coal Mine

There exists a deviation between the actual position of rod feeding and the targeted position set for the coal mine underground drilling robot. This deviation can easily lead to operational failures during rod feeding and even cause damage to the equipment of the drilling robot. To address this issue, it is necessary to correct the rod feeding position of the coal mine underground drilling robot. However, the operational range is quite extensive, and, hence, it is essential to divide the range into smaller areas. By doing so, error compensation can be implemented, thereby enhancing the automatic positioning accuracy of the coal mine underground drilling robot during rod feeding operations.

**Figure 3:**
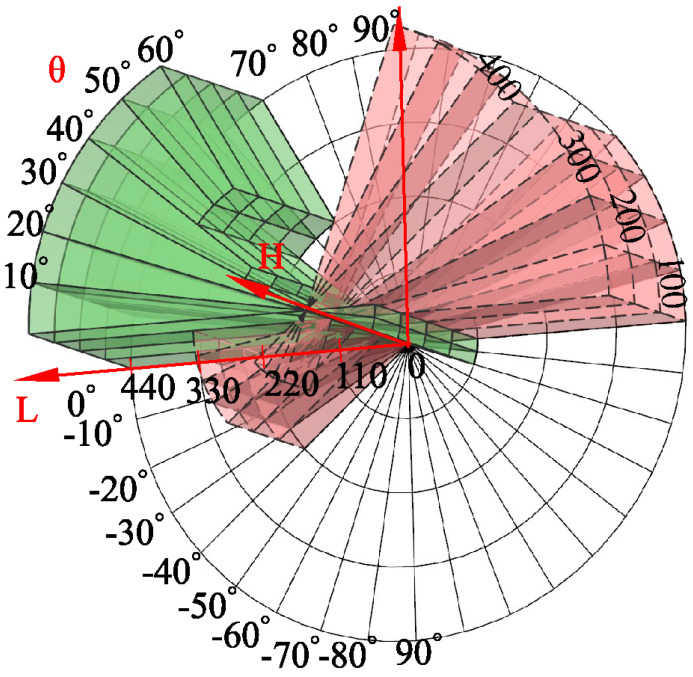
Working range of underground drilling robot in a coal mine.

Figure 3 illustrates the operational range of the coal mine underground drilling robot, with an angular range of −90° to 90°. The height direction represents the working range of the lifting column, ranging from 0 mm to 400 mm, while the cylindrical radius indicates the translational feeding device’s working range from −440 mm to 440 mm. Due to limitations imposed by factors such as the arm’s range of motion, column height, power head, roadway space, and hose placement, the working capabilities of the coal mine underground drilling robot are significantly constrained. The working range is depicted as the solid region in the figure, denoting the limited workspace.

Given the broad span of the coal mine underground drilling robot’s working range, relying solely on manual teaching methods would result in a substantial workload while offering limited improvement in rod delivery position accuracy. To enhance the precision of rod delivery, this study adopts a step-by-step division of the robot’s workspace. The workspace is divided into fan-shaped areas with an angular interval of 10° and a height interval of 100 mm. Each fan-shaped area is defined by four boundary lines in the radial direction. The points located on the boundary lines are corrected directly using the boundary error correction compensation function. Conversely, points not on the boundary lines require compensation using an anisotropic error correction algorithm based on distance and direction. Specifically, for points not on the boundary lines, the algorithm determines the corresponding division of the smallest sector workspace area based on the tilt angle, height, and translation parameters. Next, employing an error weight evaluation function that considers the distance and angle between the spatial grid’s vertex and the localization, the algorithm corrects the position of the feeder rod.

#### 3.3.2. Boundary Line Error Correction Compensation Algorithm of the Coal Mine Underground Drilling Robot

The theoretical rod delivery position of the coal mine underground drilling robot is represented by Equation (5) to Equation (8), while the target rod delivery position of the robot is obtained through manual teaching. Considering that the machining assembly error and the flexible hand claw positioning placement error are essentially fixed values, we can approximate the existence of a rotational translation matrix. This approximation allows us to align the theoretical rod delivery position with the target rod delivery position by applying coordinate translation and rotation. Consequently, the resulting theoretical rod delivery position is essentially equivalent to the target rod delivery position.

Let us assume that a set of calculations for the theoretical rod delivery position is denoted as “A”, and the corresponding target position obtained through manual teaching is denoted as “B”. Then, we have the following relationship: A → B. This implies a one-to-one correspondence between the theoretical rod delivery position and the target position. Here, the rotational translation matrix serves as the key element for reconciling the theoretical and target positions. It ensures that the effects of machining assembly errors and flexible hand claw positioning placement errors are effectively compensated for, thereby achieving a close match between the theoretical and target rod delivery positions.

(14) $$A=\left[A_{1},A_{2},A_{3}\ldots \ldots A_{n}\right]$$

1. Calculation of the center point

(15) $$cA_{i}=\sum_{j=1}^{n}\frac{A_{ij}}{n}$$

(16) $$cB_{i}=\sum_{j=1}^{n}\frac{B_{ij}}{n}$$

2. Translate the centers of A and B to the origin

(17) $$Ap=A-repmat\left(cA,\;size\left(A,1\right),\;1\right);$$

(18) $$Bp=B-repmat\left(cB,\;size\left(B,1\right),\;1\right);$$

3. Calculation of the transpose matrix of A*B

(19) U=Ap′ * Bp

4. Singular value decomposition of *U*

(20) $$\left[Ux,\;\sim ,\;Uy\right]=svd\left(U\right)$$

5. Calculation of the rotation matrix R and the translation matrix t

(21) R=Uy * Ux′

(22) t=cB′−R * cA′

6. Transformation of A

(23) $$A\_transformed=\left(R\;*\;A\prime +repmat\left(t,\;1,\;size\left(A,1\right)\right)\right)\prime$$

7. Calculation of the amount of feeder position error

(24) △A=A_transformed−A

8. Correction of the delivery position

(25) $$A_{0}=A+\Delta A$$

To obtain accurate positioning, manual teaching sampling is conducted at equal intervals along each boundary line. This process allows us to obtain the coordinates of the theoretical rod delivery position as well as the corresponding target rod delivery position. Subsequently, an error compensation function for each boundary line is derived using Equation (14) to Equation (23). Specifically, by applying the rotational translation matrix R and t, as described in Equation (23) to Equation (25), the theoretical rod delivery position A undergoes a change, resulting in a corrected theoretical rod delivery position. This correction ensures that the corrected theoretical position closely aligns with the target rod delivery position. The primary purpose of this algorithm is to compensate for errors present between boundary lines. Additionally, it establishes a foundation for compensating for errors found at points not located on the boundary lines.

#### 3.3.3. Compensation Algorithm for Error Correction in the Working Area of the Coal Mine Underground Drilling Robot

For points located on the boundary line, the boundary line error compensation function can be directly used for compensation. However, for points not situated on the boundary line, it is necessary to determine a method to correct their rod positions based on neighboring boundary line errors. Typically, the solution involves employing the inverse distance weighted spatial difference algorithm. However, this algorithm suffers from low precision in error correction due to the reliance on a single evaluation index, namely, distance. As discussed in the article, the position error vector exhibits anisotropy, meaning that neighboring positioning points not only have similar coordinates but also possess relatively close position error vectors. To address this issue, this paper proposes a spatial difference error compensation algorithm based on anisotropy.

**Figure 4:**
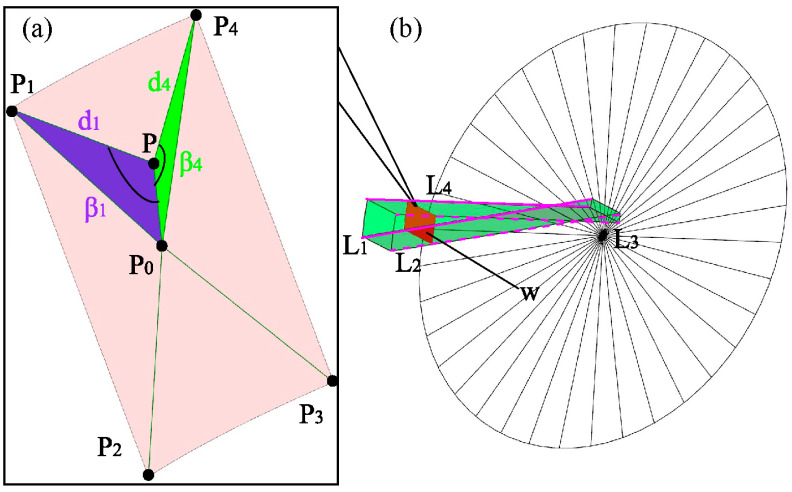
Relationship between the vertices of the spatial grid of the working range and the localization points. (**a**) Relationship between point P and the vertices (**b**) Extraction of the W sector slice where point P is located from the workspace range.

Figure 4 illustrates the relationship between the vertices of the spatial mesh within the working range and the localization points. Specifically, a cylindrical slice is formed with a radius equal to the translation *L*_1_, intersecting with the fan-shaped region to create the *W* slice. This fan-shaped slice intersects with four boundary lines, resulting in the formation of four vertices denoted as *P*_1_, *P*_2_, *P*_3_, and *P*_4_, represented as *P_i_*. These four fixed points serve to calculate the error of any localization point *P* within the fan-shaped slice. *P*_0_ represents the center of the fan-shaped slice, and the distances between *P*_0_ and *P*_1_, *P*_2_, *P*_3_, and *P*_4_ correspond to the distances between the four vertices and the localization point *P*, denoted as *d_i_*.

**Figure 5:**
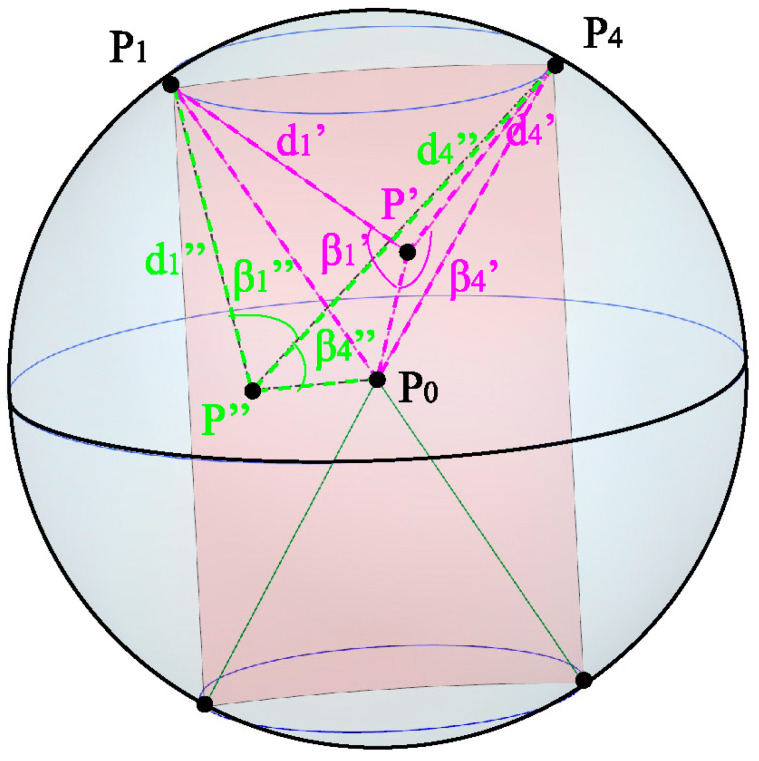
Relationship between distance and angle.

Figure 5 illustrates the relationship between the vertices of the fan-shaped sheet and the localization points. The points *P*, *P*_0_, and *P_i_* form a triangle, with *β_i_* representing the angle between the side *PP*_0_ and the side *PP_i_* of the triangle, where i ranges from 1 to 4. When point *P* is located in a different region of the fan-shaped sheet, the corresponding angle *β_i_* and the distance di undergo changes. As the locating point *P* transitions from position *P*′ to position *P*″, the corresponding angle and distance also change. Upon analyzing the figure, it is evident that as the angle increases, the distance decreases, and vice versa. In the triangle Δ*PP*_0_*P_i_*, the angle can be calculated.

(26) $$\cos(\beta_{i})=\frac{{\left|PP_{i}\right|}^{2}-{\left|P_{0}P_{i}\right|}^{2}+{\left|PP_{0}\right|}^{2}}{2\times \left|PP_{i}\right|\times \left|PP_{0}\right|},i=1,2,3,4$$

(27) $$\left|P_{0}P_{i}\right|=\sqrt{{\left(x_{0}-x_{i}\right)}^{2}+{\left(y_{0}-y_{i}\right)}^{2}+{\left(z_{0}-z_{i}\right)}^{2}}$$

(28) $$\left|P_{0}P\right|=\sqrt{{\left(x_{0}-x\right)}^{2}+{\left(y_{0}-y\right)}^{2}+{\left(z_{0}-z\right)}^{2}}$$

(29) $$\left|P_{i}P\right|=d_{i}=\sqrt{{\left(x_{i}-x\right)}^{2}+{\left(y_{i}-y\right)}^{2}+{\left(z_{i}-z\right)}^{2}}$$

The evaluation function for the weights of the positional error of the rod feeder on the coal mine downhole drilling robot, taking into account both distance and direction, can be defined as follows:

(30) $$\sigma_{i}=\eta_{1}\times \frac{{\left(\lambda_{i}\right)}^{\omega }}{\sum_{i=1}^{4}{\left(\lambda_{i}\right)}^{\omega }}+\eta_{2}\times \frac{{\left(\gamma_{i}\right)}^{\omega }}{\sum_{i=1}^{4}{\left(\gamma_{i}\right)}^{\omega }},i=1,2,3,4$$

(31) $$\lambda_{i}=\frac{1}{d_{i}}$$

(32) $$\gamma_{i}=\frac{1}{\left[\tau +\cos(\beta_{i})\right]}$$

where *ω* represents the weighted power index, typically set to a value of 1, and *τ* is also set to 1. *η*_1_ and *η*_2_ are the weighting coefficients, which determine the relative contribution of the distance evaluation function and the direction evaluation function to the overall weighting. The analysis of Equation (30) reveals that when *η*_1_ is set to 0 and *η*_2_ is set to 1, the direction evaluation function takes effect, whereas when *η*_1_ is set to 1 and *η*_2_ is set to 0, the distance evaluation function takes effect.

Therefore, the theoretical position of the rod at any given point within the sector’s working area can be calculated as *P* = (*x*, *y*, *z*). The corrected rod position is denoted as *P*″ = (*x*, *y*, *z*), and the position error of the rod is represented by Δ*P* = (Δ*x*’, Δ*y*’, Δ*z*’).

(33) $$\left\{\begin{array}{c} \Delta x\prime =\sum_{i=1}^{4}\sigma_{i}\times \Delta x_{i}\\ \Delta y\prime =\sum_{i=1}^{4}\sigma_{i}\times \Delta y_{i}\\ \Delta z\prime =\sum_{i=1}^{4}\sigma_{i}\times \Delta z_{i} \end{array}\right.$$

(34) $$\left\{\begin{array}{c} x\prime \prime =x+\Delta x\prime \\ y\prime \prime =y+\Delta y\prime \\ z\prime \prime =z+\Delta z\prime \end{array}\right.$$

After applying the anisotropy-based spatial difference error compensation algorithm, the theoretical position of the feeder is corrected, resulting in the obtained parameter coordinates *P*″ = (*x*, *y*, *z*).

## 4. Experiment

### 4.1. Experimental Platform

To evaluate the effectiveness of the anisotropy-based spatial difference error compensation algorithm proposed in this study, we conducted experiments using the ZDY4500LK coal mine underground drilling robot, provided by a specific company, as the test object. The industrial robot used in the experiments was the EFORT-ER50A, produced by EFFORT. The drilling rod employed had a diameter of φ73 mm and was manufactured by another company, with an effective length of 750 mm.

**Figure 6:**
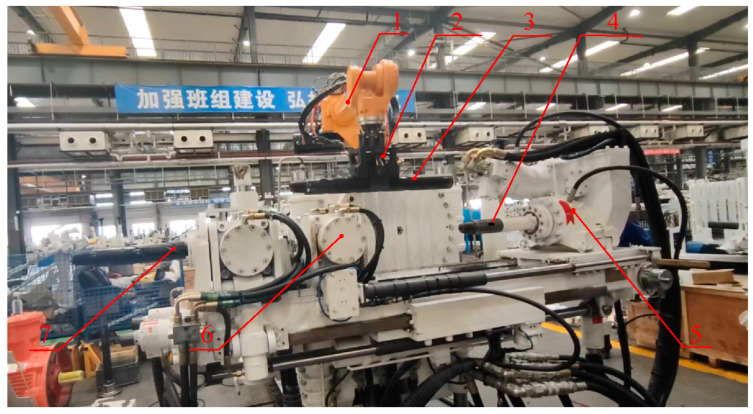
Coal mine underground drilling robot experiment platform. 1. Industrial robot; 2. flexible robotic gripper; 3. drill pipe to be connected; 4. active drill pipe; 5. power head; 6. rear gripper; 7. working drill pipe.

Figure 6 illustrates the experimental platform that was constructed for this purpose. The coal mine underground drilling robot automatically adjusted parameters such as lifting height, tilting angle, and translation amount based on the command parameters, enabling automated attitude adjustments.

**Figure 7:**
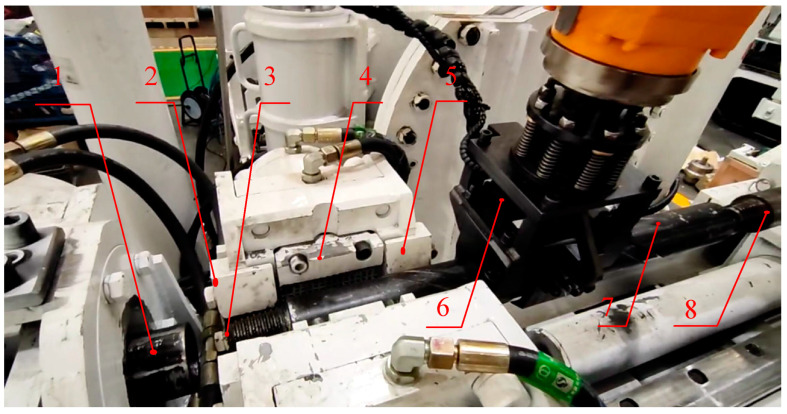
Schematic diagram of feeder rod position calibration. 1. Female joint end face of working drill pipe; 2. front face of the rear gripper; 3. male joint end face of drill pipe to be connected; 4. counter clamping cassette; 5. limiting groove; 6. flexible hand claw; 7. drill pipe to be connected; 8. male joint end face of active drill pipe.

Figure 7 shows a schematic diagram of the feed position calibration in which the industrial robot places the drill pipe to be connected to the specified position by means of a flexible manipulator. Manual teaching is used to calibrate the appropriate feeding position of the drill pipe to be connected, which requires that the drill pipe to be connected is placed between the working drill pipe and the active drill pipe, and does not interfere with the distance between the end face of the working female joint and the end face of the male joint of the active drill pipe. In order to facilitate the measurement, the front face of the drill pipe to be connected is flush with the front face of the rear gripper as the axial standard for the feeding position; the claw of the flexible manipulator and the drill pipe to be connected are perpendicular to each other (programmed by the industrial robot); during the placement, there is no interference with the rear gripper. The flexible manipulator jaws and the drill pipe to be connected are perpendicular to each other (industrial machine program control); during the placement of the drill pipe to be connected, it must not collide with the two sides and the bottom of the limit slot of the rear gripper. When the clamping chuck is clamped, the swing of the drill pipe to be connected is less than 1 degree, and the distance between the front face of the drill pipe to be connected and the front face of the rear gripper is less than 1 mm, then the manual calibration of the feeder position is considered to be successful, and the target feeder position is obtained at that point.

### 4.2. Calibration and Correction of the Pull Rope Sensor

The pull rope sensor is responsible for measuring the length of the rope in order to determine the lifting, translating, and tilting postures of the underground drilling robot. The accuracy of this sensor has a direct impact on the theoretical rod feeding accuracy and overall performance of the underground drilling robot. Therefore, it is crucial to calibrate and correct the accuracy of the pull rope sensor to minimize sensor errors and enhance the rod feeding accuracy of the underground drilling robot.

In this research paper, we employed a 5 m tape measure to measure length and a digital inclinometer with a range of 0–360° and an accuracy of 0.2° for inclination measurement. To calibrate and verify the measurement results obtained from the pull rope sensor, we used the movable limit position of the coal mine underground drilling robot as a reference point. These measurements were conducted in an environment with a room temperature of 20 °C. Each set of measurements was performed five times on average, filtering out any abnormal values, and the resulting average was taken as the final measurement result.

To illustrate the procedure, let us focus on the lift sensor. When the lift device was lowered to its lowest limit position, we recorded the number of turns measured by the rope sensor as *n*_1_, with the corresponding measured value denoted as *L*_1_. Similarly, when the lift device was raised to its highest limit position, we recorded the number of turns as *n*_2_ and the corresponding measured value as *L*_2_. We employed a tape measure to determine the distance, denoted as *L*, between the highest and lowest travel points of the lift device. Specifically, the tape measure was utilized to accurately measure the distance between the highest travel point and the lowest travel point.

Table 2 presents the calibration parameter list for the pull rope sensor, and the length coefficients for each sensor are adjusted using Equations (12) and (13). The detection results of the calibrated pull rope sensor are displayed in Table 3. The analysis reveals that the corrected pull rope sensor’s measured values are virtually identical to the actual values. Consequently, calibrating and correcting the sensors can enhance the measurement accuracy of the underground drilling robot’s sensor in coal mines.

To compensate for the position errors in the automatic pole feed, a combination of manual teaching and model correction techniques is employed. By issuing the opening position command to the coal mine underground drilling robot, the automatic posture adjustment of the drilling machine is achieved. Once the drilling robot’s opening position is fixed, the theoretical rod delivery position is calculated based on the sensor detection parameters using Equations (6)–(9). The corresponding target delivery position is determined using manual teaching methods. The boundary line rotation translation matrix is then calculated using Equations (21)–(22), followed by the calculation of the corrected position parameters for the points on the boundary line using Equation (23). Furthermore, the corrected position parameters for any point within the inner region of the boundary line are calculated using Equation (31).

### 4.3. Boundary Line Error Compensation Function

In this study, a specific sector within the working range area of the drilling robot was chosen for validation calculations. The sector encompassed lifting heights of 200 mm and 100 mm, inclination angles of 10° and 20°, and translations of −110 mm, 0 mm, 220 mm, and 440 mm using the translation device positioned along the boundary line. The results of the calculations, including the theoretical rod feed position, target rod feed position, and corrected rod feed position for the selected points on the boundary line, are presented in Table 4. Additionally, Table 5 provides the positional translation rotation matrix and the angular translation rotation matrix for the four boundary lines within the work area.

**Figure 8:**
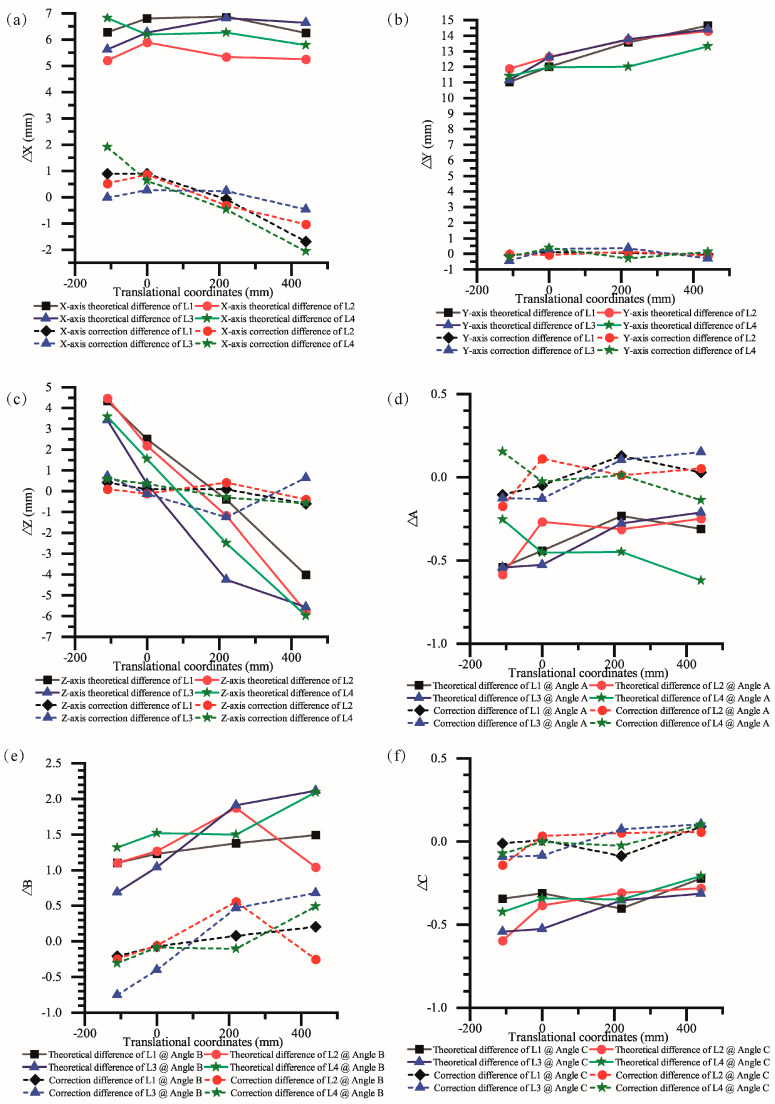
Theoretical and corrected errors of displacement and angle. (**a**) Theoretical and corrected errors in the *x*-direction; (**b**) theoretical and corrected errors in the *y*-direction; (**c**) theoretical and corrected errors in the *z*-direction; (**d**) theoretical and corrected errors of angle A; (**e**) theoretical and corrected errors of angle B; (**f**) theoretical and corrected errors of angle C.

Figure 8a–c depicts the position errors of the measured points along the four boundary lines. The theoretical error represents the disparity between the target feed position and the theoretical feed position (represented by the solid line), while the correction error shows the variance between the target feed position and the corrected feed position (dashed line). Figure 8d–f illustrates the attitude errors of the measured points on the four boundary lines.

Analyzing Figure 8a–c, it is evident that the theoretical error in the X direction ranges from 5 mm to 7 mm. However, after implementing the proposed algorithm, the corrected error is within the range of −2 mm to 2 mm. Similarly, the theoretical error in the Y direction ranges from 11 mm to 15 mm, but the corrected error now falls within −1 mm to 1 mm. In terms of the Z direction, the error prior to the correction is −6 mm to 5 mm, but it is reduced to −1 mm to 2 mm after applying the correction algorithm. Additionally, the error before the correction of angle A ranges from −0.6 mm to −0.2 mm, while the error after the correction improves to −0.2 mm to 0.2 mm. Similarly, the error before the correction of angle B is between 0.5 mm and 2 mm, but after the correction, it decreases to −0.7 mm to 0.6 mm. Lastly, the error after the correction of angle C ranges from −0.6 mm to −0.2 mm, and it is further reduced to −0.2 mm to 0.1 mm. It is observed that both the positional error and attitude error are effectively compensated through the proposed algorithm. The feed rod position and attitude are brought closer to the target position and attitude after the correction process. This indicates that the algorithm significantly enhances the accuracy of the rod position and attitude.

The translational rotation matrix for each boundary line is derived by applying Equations (21) and (22) to the theoretical parameters of the rod feed and the target rod feed parameters along each respective boundary line. The resulting translational rotation matrix for the *L_1_*~*L_4_* boundary lines is then expressed using Equation (32), yielding the following outcome:

(35) $$T=\left[\begin{array}{cc} R & t\\ 0 & 0 \end{array}\right]$$

Similarly, for the attitude correction of the drilling robot’s rod feed position (*A B C*), the calculation of the translational rotation matrix corresponding to the attitude correction of the *L*_1_~*L*_4_ boundary line can be represented by Equation (32).

### 4.4. Spatial Position Error Compensation

Additionally, in the context of spatial position error compensation, it is observed from Section 3.2 that the working range of the underground drilling robot in the coal mine is divided into multiple sector-shaped working areas. To address spatial position errors for any given point, this paper introduces a spatial difference compensation algorithm that takes into account the anisotropy of distance and direction.

**Figure 9:**
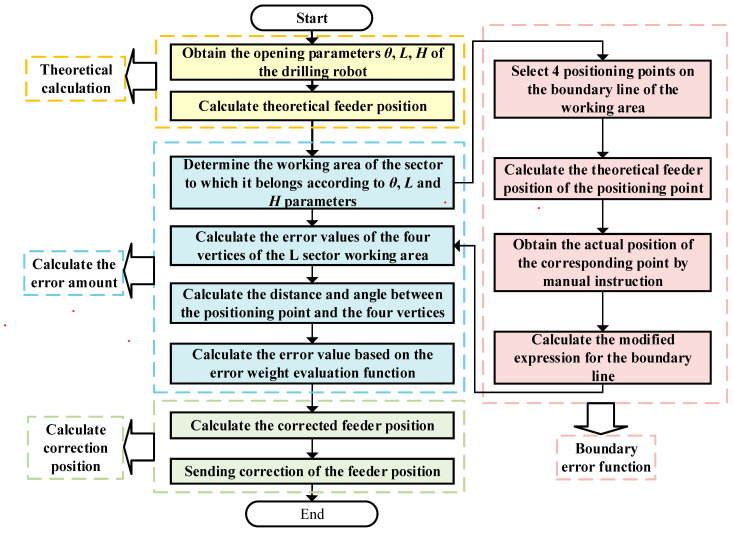
Flowchart of the feeder position correction.

Figure 9 depicts the flowchart of the anisotropy-based spatial difference compensation algorithm designed to rectify errors in the feeder position. This algorithm encompasses four essential steps: theoretical calculation, boundary error function solution, error calculation, and position correction. The first step, theoretical calculation, involves employing Equations (6)–(9) to compute the theoretical position of the rod feed based on the H Lθ parameters measured by each sensor. Subsequently, the boundary error function is determined by quantifying the discrepancy between the desired rod delivery position and the computed theoretical rod delivery position, as outlined in Equations (14)–(25). This boundary error function serves as the core element for position correction. In the subsequent step, the algorithm assigns a working area to any point in space. By employing the boundary line error function and the weight evaluation function described in Equations (26)–(33), the error value at the four vertices is computed. These calculations enable the determination of the error magnitude at the positioning point. Lastly, Equation (34) is employed to correct the position of the feeder rod, resulting in the final adjusted position.

In Table 6, the positional errors of vertices *P*_1_, *P*_2_, *P*_3_, and *P*_4_ are presented for the given values of *θ* = 17.05°, *L* = 280.51 mm, and *H* = 183.03 mm. Initially, the theoretical values of points *P*_1_–*P*_4_ are derived using the positional parameters. Subsequently, the corresponding *L*_1_–*L*_4_ parameters are corrected to obtain the corrected values, and the error magnitudes for each vertex are computed by subtracting the theoretical values from the corrected parameters.

Table 7 indicates the amount of attitude error of the four vertices of the fan-shaped work-piece body belonging to point *P* at points *P*_1_, *P*_2_, *P*_3_, and *P*_4_ for *θ* = 17.05°, *L* = 280.51 mm, and *H* = 183.03 mm.

Table 8 presents the weights assigned to the evaluation functions, which are calculated separately for the direction evaluation function (*η*_1_ = 0, *η*_2_ = 1), used to correct positional parameters, and the distance evaluation function (*η*_1_ = 1, *η*_2_ = 0), used to correct positional parameters. This comparison aims to assess the impact of these two evaluation functions on the correction results. An analysis of Table 7 reveals that, when using the distance evaluation function, even if the points are in close proximity to the reference point, the assigned weights are not significantly higher compared to the weights assigned to other vertices. Consequently, the similarity of the errors may not be effectively captured, and the relatively high weights assigned to other vertices can adversely affect the interpolation accuracy of the reference point. Conversely, the direction evaluation function solely takes positional similarity into account while disregarding the influence of other vertices on the results. To address this limitation, this paper selects *η*_1_ = 0.5 and *η*_2_ = 0.5 as the parameters to enhance the accuracy of the differences between evaluated points.

**Figure 10:**
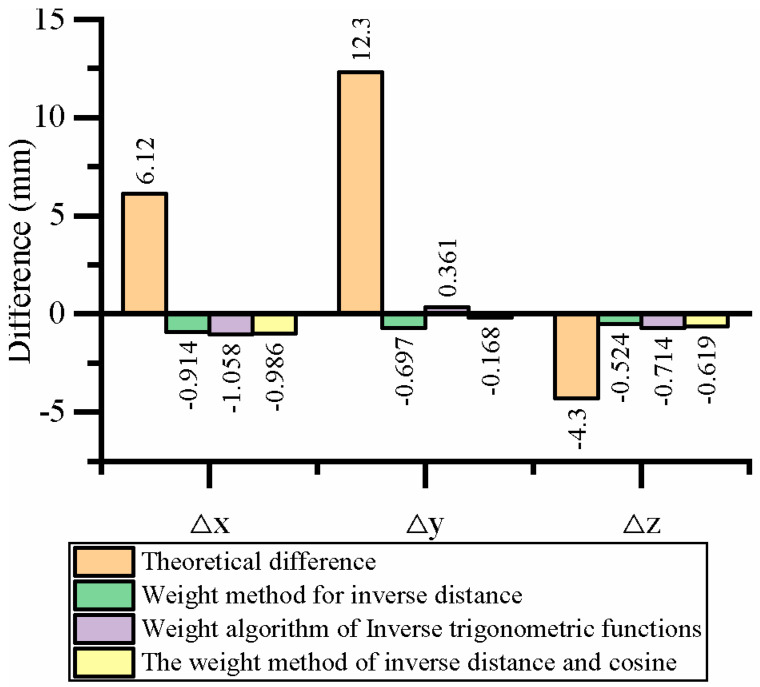
Comparison of positional differences after the correction.

**Figure 11:**
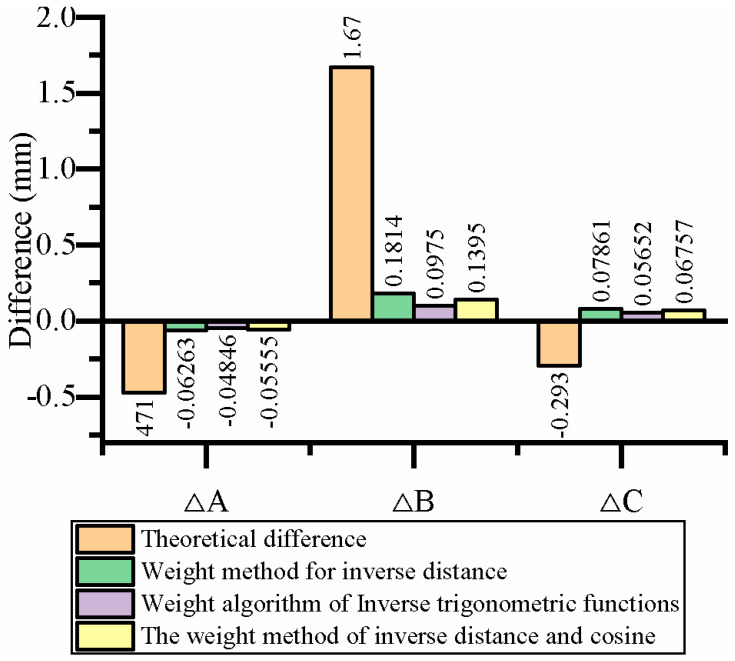
Comparison of angular differences after the correction.

Table 9 presents a comparison of the results obtained from distance correction, direction correction, and distance and direction correction methods for position correction. Figure 10 illustrates the graph depicting the differences in corrected positions. Additionally, Table 10 displays a comparison of the results achieved from distance correction, direction correction, and distance and direction correction methods for angle correction. Figure 11 visually represents the differences in corrected angles. Based on the analysis, it is evident that the theoretical position and angle deviations of the rod delivery were relatively large before the correction process. However, the application of distance correction, direction correction, and distance and direction correction methods all result in a significant reduction in the position error of the rod delivery. Among them, the distance and direction correction method produces more balanced results compared to the distance correction and direction correction methods alone. This improvement can be attributed to the utilization of an anisotropic spatial difference compensation algorithm, which simultaneously considers both distance and direction weights. As a result, the overall correction outcomes are improved.

Table 11 presents a comparison of the effects of different correction methods on correcting multiple arbitrary points. Upon analyzing Table 11, it is observed that for the same positioning point, the distance evaluation function, direction evaluation function, and distance and direction evaluation function are utilized to compensate for differences. The theoretical error is found to be relatively large in both position and attitude aspects. Upon applying the distance evaluation function and the direction evaluation function for compensation, the differences in error are not significant. However, the distance evaluation function yields better results for specific point positions or angles compared to the direction evaluation function. Conversely, the direction evaluation function achieves superior results for certain point positions or angles compared to the distance evaluation function. This indicates that for positioning points in the workspace with better error similarity between the point location and the vertex, the direction evaluation function leverages the advantage of error similarity. On the other hand, for general points with limited error similarity, employing the distance evaluation function facilitates a more even weight distribution. Using the direction evaluation function in such cases amplifies the error, which contradicts the objective of achieving minimal fluctuations in the rod delivery position error. To address this concern, the anisotropic spatial difference compensation algorithm is employed to mitigate error volatility. Experimental results demonstrate that the error correction algorithm based on distance and direction is universally applicable and can be employed for compensating rod feeding positions at any point within the workspace. This enhancement significantly improves the rod feeding accuracy of the coal mine drilling robot.

Based on Equations (30)–(32), it is clear that the difference value of point P remains constant for both the distance evaluation function and the direction evaluation function when considering points located on the boundary line. Therefore, when dealing with boundary line points, the computational results are identical whether the conventional inverse distance-weighted spatial difference algorithm or the spatial difference compensation algorithm proposed in this paper, which incorporates the anisotropy of distance and direction, are used. For any point within the operational range that is not on the boundary line, the error correction algorithm suggested in this paper, which considers both distance and direction, outperforms distance-only or direction-only based correction approaches by significantly reducing positional and angular errors of the feeder rod. This algorithm ensures a more stable and effective compensation, thereby enhancing the overall performance of the system.

## 5. Field Tests

### 5.1. Construction

**Figure 12:**
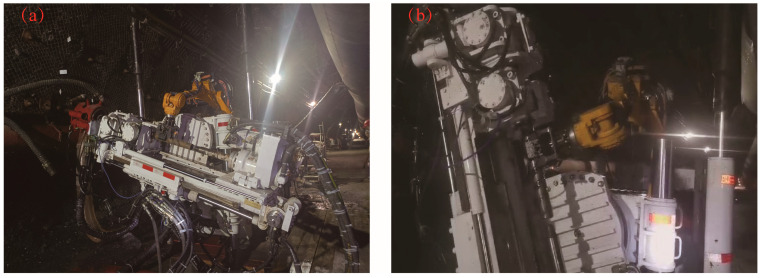
Site plan for drilling construction. (**a**) Site plan for small angle drilling; (**b**) site plan for large angle drilling.

Figure 12 represents a diagram of the drilling site at different inclination angles. In the 21,136 roadway of a mine owned by the Huainan Mining Group, an underground drilling robot was successfully deployed to construct a pressure relief hole that traverses multiple layers in the bottom drawway. The primary stratum consists of mudstone, with a hardness coefficient ranging from 0.5 to 1. The average thickness of the 6th coal seam measured 2.5 m, while the 8th coal seam had an average thickness of 3.3 m. During the test, a ribbed drill pipe with a diameter of Ø 73 mm and an effective length of 750 mm was employed, along with a drill bit measuring Ø 113 mm in diameter. Water was supplied to the rock section holes, while air was utilized for the coal section holes throughout the entire construction process. In total, seven drill holes were successfully completed.

Table 12 presents the drilling parameters utilized during the construction of an underground coal mine by the drilling robot. A total of seven drill holes were successfully completed, with depths ranging from 50 to 115 m. Throughout the operation, the rods were loaded and unloaded 737 times each. However, an abnormality was observed during the construction of hole No. 4-6-4, where the angle of the rod feeding position deviated significantly. Upon analysis, it was determined that this deviation was caused by inaccurate measurements from the inclination sensor. Due to the narrow roadway in the underground coal mine, the drilling robot had to be disassembled into two parts for transportation and then reassembled on-site. Unfortunately, the zero-point calibration of the inclination sensor was not performed after reassembly, resulting in an incorrect rod feeding position. Furthermore, during the construction of hole 4-8-9, another abnormality occurred due to a relatively large opening inclination angle of 71.1°. This anomaly was primarily caused by water ingress inside the panning sensor, leading to incorrect measurements and subsequent miscalculation of the rod delivery position. Apart from these two anomalies, the automatic rod feeding position of underground drilling robots in the remaining holes was normal, meeting all requirements for site construction.

### 5.2. Experimental Insights

The automated rod feeding operation of the underground coal mine drilling robot is designed to meet construction requirements. However, during practical usage, the challenging working environment in underground coal mines and the occurrence of abnormal issues have provided valuable insights through testing and experience. These insights can be summarized as follows:

1. It is crucial to conduct sensor zero calibration during the disassembly and assembly of the coal mine drilling robot to ensure the precise measurement of all sensors. Meticulous verification of the sensor measurement results is necessary to ensure their accuracy.
2. All sensors must possess high waterproof, dustproof, and anti-vibration capabilities. When drilling, the expulsion of cinders, water, and other impurities from the hole can directly impact the sensors, potentially causing damage. Such damage can impede the accurate collection of crucial parameters associated with the automatic feeder’s position, thereby compromising the overall accuracy of the automatic feeder.
3. Whenever new sensors are installed, it is essential to perform coefficient correction, zero calibration, and accuracy verification of the measurement results. These steps are necessary to ensure the precise determination of the automatic rod’s position.

## 6. Conclusions

In this study, we have proposed a positioning error compensation algorithm based on modeling and manual teaching for the coal mine underground robot. Firstly, we established a theoretical model for the rod feeding position through spatial coordinate system conversion and spatial position relationship. Additionally, we analyzed the potential factors contributing to the significant rod feeding error. We identified that the measurement error can be mitigated by correcting the sensor error, while the manipulator processing error and flexible manipulator can be compensated and rectified through manual teaching. To address boundary line error, we divided the working area into several sectors based on the step size and developed a compensation algorithm utilizing the translation rotation matrix for boundary line error correction. Furthermore, we proposed a spatial difference compensation algorithm based on anisotropy, taking into account the spatial geometric relationship. Finally, we validated the effectiveness of the proposed method through experimental comparisons. In summary, our study provides a comprehensive approach to tackling positioning errors in the coal mine underground robot. By integrating modeling, manual teaching, and compensation algorithms, we have significantly improved the accuracy of rod feeding. The proposed algorithms successfully address sensor errors, manipulator processing errors, and flexible manipulator uncertainties, leading to enhanced performance and efficiency in coal mine drilling operations.

Compared to other papers, this paper introduces several notable innovations in the field of coal mine underground drilling. These innovations are outlined as follows:

1. The establishment of a theoretical model for the feeder rod position of an underground coal mine drilling robot is the first-ever achievement in this domain. This model is based on the transformation relationship of the spatial coordinate system.
2. This paper proposes a unique approach to meet the challenge of conventional equipment and techniques that cannot be used underground in coal mines due to explosion-proof requirements. This innovative method combines the theoretical model with manual teaching techniques to overcome the issue of poor accuracy in the automatic rod feeding position of the drilling robot. It brings forth simplicity, feasibility, and reliability.
3. In order to effectively address the problem of a large working range and insufficient compensation capability of the underground coal mine drilling robot, the robot integrates the practical usage scenario into its design. It divides the working area into multiple smaller units and introduces an anisotropic spatial difference compensation algorithm that incorporates both distance and direction. This approach significantly enhances the accuracy of the automatic rod feeding position.

Experimental results demonstrate that the anisotropic spatial difference compensation algorithm, based on the theoretical model and manual teaching, significantly enhances the accuracy of the rod feeding position. Moreover, it fulfills the requirements for practical field use. To facilitate the mass production of drilling robots in underground coal mines, it is recommended to simplify the subsequent operation process and solidify the operation steps. This ensures that the automatic rod feeding and positioning correction compensation algorithm is reproducible and standardized.

## Figures and Tables

**Table 1:** Nominal values of parameters between the components of the ZDY4500LK coal mine underground drilling robot.

Part Number *i*	*x*-axis Distance *a_i_* (mm)	*y*-axis Distance *b_i_* (mm)	*z*-axis Distance *c_i_* (mm)	Angle *α_i_* (°)	Angle *β_i_* (°)	Angle *γ_i_* (°)
1	0	0	0	0	0	0
2	0	1345	240	0	0	0
3	0	0	300 + *H*	0	*θ*	0
4	−1100 + *L*	0	−178	0	0	0
5	897 + *S*	0	178	0	0	0

**Table 2:** Calibration parameters for pull cord sensors.

Name	Cord Sensor Reading at Minimum Limit Position *n*_1_	Measured Value of the Minimum Limit Position *L*_1_	Cord Sensor Reading at Maximum Limit Position *n*_2_	Measured Value of the Maximum Limit Position *L*_2_	Theoretical Measurements *L* = *L*_2_*-L*_1_	Actual Measured Value *L’*	Theoretical Length Factor *K*	Actual Length Factor *K’*
Lift Sensor	1	0.049	8282	404.395	404.346	400	0.048828125	0.048303345
Panning Sensor	−9100	−444.336	9101	444.385	888.721	880	0.048828125	0.048348992
Angle Sensor	0	0.000	1043	91.670	91.670	90	0.087890625	0.086289549

**Table 3:** Detection results of calibrated pull rope sensors.

Name	Sensor Count	K before Correction	Corrected K	Actual Measured Value	Results before Correction	Pre-Correction Error	Corrected Result	Corrected Error
Lift Sensor	4151	0.048828125	0.048303345	200.5	202.686	−2.186	200.5072	−0.007
Panning Sensor	2068	0.048828125	0.048348992	100	100.977	−0.977	99.9857	0.014
Angle Sensor	116	0.087890625	0.086289549	10	10.195	−0.195	10.010	−0.010

**Table 4:** Statistics of the theoretical, measured, and modified parameters of the boundary line.

Name	Number	Height (mm)	Translation (mm)	Angle (°)	Theoretical Parameter						Measured Parameter						Corrected Parameter					
					*X* (mm)	*Y* (mm)	*Z* (mm)	*A* (°)	*B* (°)	*C* (°)	*X* (mm)	*Y* (mm)	*Z* (mm)	*A* (°)	*B* (°)	*C* (°)	*X* (mm)	*Y* (mm)	*Z* (mm)	*A* (°)	*B* (°)	*C* (°)
*L*_1_	1	100.02	−110.7	20.04	−1.445	1345	916.25	0	159.96	−90	4.839	1356.033	920.587	−0.541	161.062	−90.345	3.952	1356.1	920.18	−0.435	161.27	90.333
	2	99.9	0.69	19.93	103.8	1345	954.1	0	160.07	−90	110.607	1357.016	956.616	−0.44	161.299	−90.312	109.72	1356.9	956.52	−0.39	161.37	−90.322
	3	99.81	219.93	19.86	310.39	1345	1028.4	0	160.14	−90	317.272	1358.564	1028.001	−0.233	161.516	−90.403	317.35	1358.5	1027.9	−0.36	161.44	−90.316
	4	99.81	439.9	19.81	517.68	1345	1102.6	0	160.19	−90	523.934	1359.655	1098.59	−0.312	161.683	−90.222	525.63	1359.7	1099.2	−0.34	161.48	−90.311
*L*_2_	5	100.24	−110.2	10.12	46.656	1345	912.68	0	169.88	−90	51.864	1356.889	917.154	−0.586	170.983	−90.596	51.354	1356.9	917.06	−0.411	171.23	−90.455
	6	100.24	0.19	10.04	155.74	1345	931.86	0	169.96	−90	161.636	1357.639	934.045	−0.269	171.23	−90.384	160.78	1357.7	934.16	−0.379	171.29	−90.418
	7	100.12	220.23	9.91	373.15	1345	969.25	0	170.09	−90	378.497	1358.735	968.077	−0.314	171.96	−90.308	378.82	1358.6	967.67	−0.325	171.4	−90.359
	8	100	439.9	9.86	589.87	1345	1006.4	0	170.14	−90	595.119	1359.291	1000.608	−0.251	171.181	−90.28	596.16	1359.4	1001	−0.304	171.43	−90.336
*L*_3_	9	200	−110	10.13	46.805	1345	1012.5	0	169.87	−90	52.431	1356.15	1015.908	−0.542	170.559	−90.542	52.449	1356.6	1015.2	−0.415	171.31	−90.451
	10	200	0.2	10.04	155.75	1345	1031.6	0	169.96	−90	162.016	1357.602	1031.959	−0.526	171.002	−90.525	161.76	1357.3	1032.1	−0.397	171.4	−90.439
	11	199.91	220.16	9.96	353.1	1345	1065.9	0	170.04	−90	359.919	1358.771	1061.655	−0.278	171.952	−90.354	359.69	1358.4	1062.9	−0.381	171.48	−90.428
	12	199.86	439.9	9.88	589.74	1345	1106.5	0	170.12	−90	596.383	1359.405	1100.936	−0.213	172.237	−90.313	596.85	1359.7	1100.3	−0.365	171.56	−90.417
*L*_4_	13	199.91	−110.4	20.06	−1.259	1345	1016.2	0	159.94	−90	5.5666	1356.409	1019.789	−0.255	161.256	−90.425	3.6645	1356.6	1019.2	−0.409	161.56	−90.352
	14	199.91	0.4	20	103.15	1345	1054.1	0	160	−90	109.348	1356.979	1055.656	−0.453	161.523	−90.343	108.72	1356.6	1055.3	−0.428	161.61	−90.34
	15	199.82	219.91	19.91	310.03	1345	1128.6	0	160.09	−90	316.304	1357.011	1126.103	−0.447	161.589	−90.349	316.77	1357.3	1126.4	−0.458	161.69	−90.323
	16	199.82	439.9	19.84	517.44	1345	1202.9	0	160.16	−90	523.226	1358.321	1196.911	−0.62	162.252	−90.207	525.29	1358.2	1197.5	−0.48	161.76	−90.309

**Table 5:** Parameters of the translation rotation matrix.

Number	Name	*r*_11_	*r*_12_	*r*_13_	*r*_21_	*r*_22_	*r*_23_	*r*_31_	*r*_32_	*r*_33_	*t*_1_	*t*_2_	*t*_3_
Displacement	*L*_1_	0.95475	0.26253	0.13974	−0.27076	0.57288	0.77363	0.12304	−0.77646	0.61804	−475.81	−123.61	1398.4
	*L*_2_	0.99179	0.1104	0.064607	−0.12216	0.66768	0.73436	0.037936	−0.73622	0.67568	−202.37	−205.64	1288.8
	*L*_3_	0.94944	−0.062633	0.30764	−0.057987	0.92805	0.36791	0.30855	0.36715	−0.8775	−219.23	−261.46	1395.4
	*L*_4_	0.94415	0.28176	0.17083	0.2963	−0.4992	−0.81425	0.14414	−0.8194	0.55481	−547.71	2855.8	1557.7
Angle	*L*_1_	−0.91176	0.41073	0	0.40831	0.90637	−0.10852	0.044574	0.098947	0.99409	−66.135	6.5219	−16.692
	*L*_2_	0.90648	0.41313	−0.087285	−0.40325	0.7857	−0.46911	−0.12522	0.46043	0.87882	−78.45	−4.464	−89.58
	*L*_3_	−0.97998	0.19909	0	0.19709	0.97016	−0.14125	0.028121	0.13842	0.98997	−34.234	−6.199	−24.867
	*L*_4_	−0.94583	−0.32466	0	−0.31772	0.92561	−0.20569	−0.066779	0.19455	0.97862	51.517	−4.998	−33.392

**Table 6:** Position error of points *P*_1_, *P*_2_, *P*_3_, and *P*_4_ at *θ* = 17.05°, *L* = 280.51 mm, and *H* = 183.03 mm.

Name	*P*_1_			*P*_2_			*P*_3_			*P*_4_		
Position coordinate	X	Y	Z	X	Y	Z	X	Y	Z	X	Y	Z
Theoretical value (mm)	363.55	1345	1049	429.05	1345	979.66	429.05	1345	1079.7	363.55	1345	1149
Matrix correction (mm)	370.98	1360	1047.1	434.94	1359.4	976.8	436.04	1359.1	1074.2	370.79	1356.5	1145.5
Difference (mm)	7.43	15	−1.9	5.89	14.4	−2.86	6.99	14.1	−5.5	7.24	11.5	−3.5

**Table 7:** Angular errors of points *P*_1_, *P*_2_, *P*_3_, and *P*_4_ at θ = 17.05°, *L* = 280.51 mm, and *H* = 183.03 mm.

Name	*P*_1_			*P*_2_			*P*_3_			*P*_4_		
Position coordinate	*A*	*B*	*C*	*A*	*B*	*C*	*A*	*B*	*C*	*A*	*B*	*C*
Theoretical value (mm)	0	160	−90	0	170	−90	0	170	−90	0	160	−90
Matrix correction (mm)	−0.4182	161.31	−90.329	−0.36225	171.32	−90.401	−0.3887	171.44	−90.433	−0.4286	161.61	−90.34
Difference (mm)	−0.4182	1.31	−0.329	−0.36225	1.32	−0.401	−0.3887	1.44	−0.433	−0.4286	1.61	−0.34

**Table 8:** Weights of the evaluation function.

Name	Distance to Positioning Points Di (mm)	Weights of the Distance Evaluation Function ui	Value of Cosine cos(bi)	Weights of the Directional Evaluation Function hi
1	84.276	0.1264	0.528	0.0370
2	89.91	0.1185	0.501	0.0352
3	38.442	0.2771	1.193	0.0837
4	22.283	0.4780	12.035	0.8441

**Table 9:** Comparison of results for various correction positions.

Name	Theoretical Value	Measured Value	Theoretical Difference	Distance Correction	Distance Correction Difference	Directionality Amendment	Directionally Corrected Differentials	Direction + Distance Correction	Direction + Distance Error
*X*	384.111	390.231	6.12	391.145	−0.914	391.289	−1.058	391.217	−0.986
*Y*	1345	1357.31	12.31	1358.007	−0.697	1356.949	0.361	1357.478	−0.168
*Z*	1112.8	1108.5	−4.3	1109.024	−0.524	1109.214	−0.714	1109.119	−0.619

**Table 10:** Comparison of results of various correction angles.

Name	Theoretical Value	Measured Value	Theoretical Difference	Distance Correction	Distance Correction Difference	Directionality Amendment	Directionally Corrected Differentials	Direction + Distance Correction	Direction + Distance Error
*A*	0	−0.471	−0.471	−0.40837	−0.06263	−0.42254	−0.04846	−0.41545	−0.05555
*B*	162.95	164.622	1.672	164.4406	0.1814	164.5245	0.0975	164.4825	0.1395
*C*	−90	−90.293	−0.293	−90.37161	0.07861	−90.34952	0.05652	−90.36057	0.06757

**Table 11:** Effect of different correction methods for multiple arbitrary points.

Number	Height (mm)	Translation (mm)	Angle (°)	Name	*X*	*Y*	*Z*	*A*	*B*	*C*
					(mm)	(mm)	(mm)	(°)	(°)	(°)
1	130.52	−96.3	12.01	Theoretical value	48.307	1345	946.615	0	167.99	−90
				Measured value	54.228	1357.592	951.588	−0.506	169.188	−90.556
				Theoretical difference	5.921	12.592	4.973	−0.506	1.198	−0.556
				Distance correction	53.446	1356.478	950.281	−0.395	169.371	−90.373
				Distance Correction Difference	0.782	1.114	1.307	−0.111	−0.183	−0.183
				Directionality amendment	53.054	1356.661	950.858	−0.365	169.312	−90.398
				Directionally corrected differentials	1.174	0.931	0.73	−0.141	−0.124	−0.158
				Direction + distance correction	53.25	1356.57	950.57	−0.38	169.341	−90.385
				Direction + distance error	0.978	1.022	1.018	−0.126	−0.153	−0.171
2	160.32	130.09	14.55	Theoretical value	255.153	1345	1035.159	0	165.45	−90
				Measured value	261.166	1358.679	1035.459	−0.399	166.852	−90.372
				Theoretical difference	6.013	13.679	0.3	−0.399	1.402	−0.372
				Distance correction	261.395	1357.752	1034.297	−0.401	166.89	−90.378
				Distance Correction Difference	−0.229	0.927	1.162	0.002	−0.038	0.006
				Directionality amendment	261.574	1357.885	1033.199	−0.394	166.911	−90.42
				Directionally corrected differentials	−0.408	0.794	2.26	−0.005	−0.059	0.048
				Direction + distance correction	261.486	1357.818	1033.748	−0.398	166.901	−90.399
				Direction + distance error	−0.32	0.861	1.711	−0.001	−0.049	0.027
3	190.54	401.12	19.51	Theoretical value	480.716	1345	1176.702	0	160.49	−90
				Measured value	486.513	1358.126	1170.911	−0.603	162.428	−90.235
				Theoretical difference	5.797	13.126	−5.791	−0.603	1.938	−0.235
				Distance correction	488.409	1356.636	1171.166	−0.419	162.037	−90.353
				Distance Correction Difference	−1.896	1.49	−0.255	−0.184	0.391	0.118
				Directionality amendment	488.587	1356.54	1171.182	−0.426	162.081	−90.344
				Directionally corrected differentials	−2.074	1.586	−0.271	−0.177	0.347	0.109
				Direction + distance correction	488.498	1356.588	1171.174	−0.422	162.059	−90.349
				Direction + distance error	−1.985	1.538	−0.263	−0.181	0.369	0.114

**Table 12:** Drilling parameters during field construction for the underground drilling robots in the coal mines.

Number	Hole Number	Drill Opening Angle (°)	Design Depth of Drill Holes (m)	Actual Depth of Drill Hole (m)	Number of Drill Pipes	Design See Coal No.	Designed Depth of Coal Sighting and Coal Stopping (m)	Actual Depth of Coal Sighting and Stopping (m)	Normal or Not
1	4-6-4	19	52.3	53.5	71	6th coal	44.3–52.3	44.6–53	Exceptional
2	4-6-5	12	71.1	72.5	97	6th coal	60.1–71.1	61.5–72	Normal
4	4-8-9	71.2	66	64.5	86	8th coal	48.7–52.5	60.2–64.2 m	Exceptional
5	4-8-10	54.1	69.76	71.75	96	8th coal	66–69	65–71.3	Normal
3	4-8-11	47	79	81.5	109	8th coal	75–79	77–81	Normal
6	4-8-12	38	92	94.5	126	8th coal	87–92	88.5–94	Normal
7	4-8-13	31	106.4	114.75	153	8th coal	100.8–106.4	108–114.25	Normal

## Data Availability

The data presented in this paper are available after contacting the corresponding author.
